# Decreased postnatal neurogenesis in the hippocampus combined with stress experience during adolescence is accompanied by an enhanced incidence of behavioral pathologies in adult mice

**DOI:** 10.1186/1756-6606-1-22

**Published:** 2008-12-17

**Authors:** Fumihiko Hayashi, Noriko Takashima, Akiko Murayama, Kaoru Inokuchi

**Affiliations:** 1 Mitsubishi Kagaku Institute of Life Sciences, MITILS, 11 Minamiooya, Machida, Tokyo 194-8511, Japan; 2 Japan Science and Technology Agency, CREST, Kawaguchi, Saitama 332-0012, Japan; 3 Graduate School of Environment and Information Sciences, Yokohama National University, Yokohama, Kanagawa 240-8501, Japan

## Abstract

**Background:**

Adolescence is a vulnerable period in that stress experienced during this time can affect the incidence of psychiatric disorders later, during adulthood. Neurogenesis is known to be involved in the postnatal development of the brain, but its role in determining an individual's biological vulnerability to the onset of psychiatric disorders has not been addressed.

**Results:**

We examined the role of postnatal neurogenesis during adolescence, a period between 3 to 8 weeks of age in rodents. Mice were X-irradiated at 4 weeks of age, to inhibit postnatal neurogenesis in the sub-granule cell layer of the hippocampus. Electrical footshock stress (FSS) was administered at 8 weeks old, the time at which neurons being recruited to granule cell layer were those that had begun their differentiation at 4 weeks of age, during X-irradiation. X-irradiated mice subjected to FSS during adolescence exhibited decreased locomotor activity in the novel open field, and showed prepulse inhibition deficits in adulthood. X-irradiation or FSS alone exerted no effects on these behaviors.

**Conclusion:**

These results suggest that mice with decreased postnatal neurogenesis during adolescence exhibit vulnerability to stress, and that persistence of this condition may result in decreased activity, and cognitive deficits in adulthood.

## Background

In studies of the etiology of psychiatric disorders, the biological vulnerability-stress hypothesis is well known by those interested in elucidating the onset of schizophrenia [1]. Gene-environment interactions are prominent within a variety of disorders. Genetic factors can confer biological vulnerability, and in such affected individuals, relatively weak environmental factors such as stress can trigger the onset of psychiatric disorders [2]. Major depression is one such disorder, in which genetic factors influence the risk of illness and the sensitivity of individuals to the depressogenic effects of environmental adversity [3]. In disorders related to social behavior, the role of serotonin transporter genotype is thought to be key [4]. However, the actual mechanisms conferring biological vulnerability to various disorders are unclear, despite the number of studies reporting associations between particular genes and symptoms of psychiatric disorders.

Adolescent brain development is a physiologically important process. Adolescence is a period bringing both vulnerability to stress and opportunities to alleviate adverse effects of events experienced earlier in development [5]. Although few experiments have examined the effects of stress on the structure of the pubertal brain, it is widely recognized that stressors experienced during adolescence can have long-lasting and profound consequences for the future behavioral and psychological function of an individual [6]. Adolescence is considered as vulnerable period during which stress can induce dysfunction of the prefrontal cortex. In addition, dysfunction of the neurobiological factors involved in adolescent change can increase the individual's susceptibility to impaired judgment, drug addiction and psychiatric disorders [7]. In rodents, the period between postnatal days 21 and 60 is taken to be equivalent to adolescence, although this is influenced by the maturational index [8].

Postnatal development of neural circuits in the forebrain is important for normal physiological function [9]. Postnatal neurogenesis is known to occur in the forebrain [10]. Because aversive stress can inhibit neurogenesis [11], neurogenesis in response to environmental factors might impact upon behavior. Impaired prenatal neurogenesis has been reported to result in abnormal behavior later, during adulthood [12]. However, it remains to be seen whether unusual neurogenesis during adolescence increases vulnerability to stress and results in abnormal behavior in adulthood.

We hypothesized that abnormalities in postnatal neurogenesis might influence biological vulnerability for the onset of psychiatric disorders, and more specifically, that decreased neurogenesis in the hippocampus might cause various behavioral. To examine the role of neurogenesis during adolescence, mice were X-irradiated at 4 weeks of age, and were subjected to electrical footshock stress (FSS) at 8 weeks. Behavioral changes relevant to psychiatric disorders were examined at 20 weeks of age. Behavioral changes such as decreased activity in the novel open field, and deficits in sensory-gating were observed in adulthood. X-irradiation or FSS alone had no effect on the onset of abnormal behaviors. These findings suggest that abnormal postnatal neurogenesis may be involved in the incidence of psychiatric disorder-related recognition deficits.

## Results

**Figure 1:**
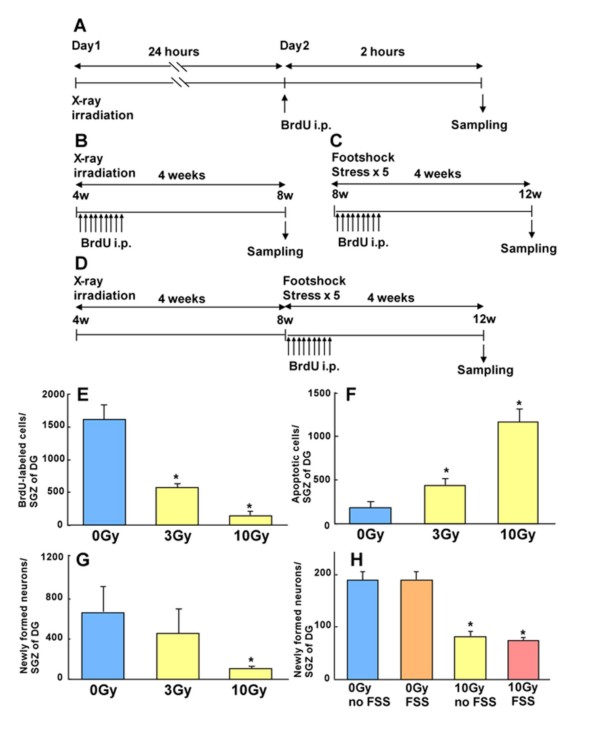
**X-irradiation inhibited cell proliferation and increased cell death in the dentate gyrus of the hippocampus, and decreased numbers of newly-formed neurons**. (A) Mice were X-irradiated at 4 weeks of age, were injected with BrdU (50 mg/kg body weight) one day later, and were then sacrificed after 2 hours. Cell proliferation and cell death were examined in the dentate gyrus of hippocampus. To estimate levels of neurogenesis, (B) we administered BrdU (50 mg/kg body weight, three times a day) from the day after irradiation for three consecutive days. 4 weeks after the last injection, mice were sacrificed and processed for histological analysis. To analyse the effects of stress upon neurogenesis, (C) mice were injected with BrdU at 8 weeks of age after 5 consecutive days of footshock stress, and were then sacrificed at 12 weeks of age. (D) To examine the synergistic effects of stress and X-irradiation, mice were irradiated at 4 weeks of age and treated using the same procedure as described in (C). (E) Strong inhibition of cell proliferation was observed in the 10 Gy-irradiated group compared to the 0 Gy group. (F) Cell death was estimated using the TUNEL method. 10 Gy irradiation caused significant cell death n comparison to 0 Gy. (G) Using immunohistochemistry with anti-BrdU and anti-NeuN antibodies, a significant decrease in the number of newly-formed neurons was observed following 10 Gy irradiation. (H) No inhibitory effects were observed as a result of the stress induction technique used in this study, and nor was any synergistic effect irradiation and stress found. All data were expressed as mean ± S.D., n = 4–6.

Mice were X-irradiated at 4 weeks of age and the effects on hippocampal neurogenesis were examined (Additional file 1, Fig 1A, 1B). Mice were injected with BrdU one day after X-irradiation, sacrificed 2 hours later and the brain tissue sections immunostained using an anti-BrdU antibody (Fig 1A). Numbers of BrdU positive cells in the sub-granule cell layer of the hippocampus were decreased in the X-irradiated mice in a dose-dependent manner (0 Gy: 1615 ± 221; 3 Gy: 570 ± 60; 10 Gy: 141 ± 65 cells/hemi dentate gyrus; Fig 1E). It was found that 10 Gy irradiation caused cell death in dentate gyrus of hippocampus, as determined using TUNEL staining (0 Gy: 186 ± 74; 3 Gy: 444 ± 74; 10 Gy: 1162 ± 145 cells/hemi dentate gyrus; Fig 1A, 1F). These results showed that X-irradiation arrested the proliferation of stem/progenitor cells and was accompanied by cell death. We next examined changes in the number of newly formed neurons following X-irradiation, occurring as a result of the inhibition of cell proliferation (Fig 1B). BrdU was administered following X-irradiation and brain sections were prepared 4 weeks later, when mice had reached 8 weeks of age (Fig 1B). Immunostaining with anti-BrdU and anti-NeuN antibodies revealed that 10 Gy of irradiation significantly decreased numbers of newly formed neurons (0 Gy: 667 ± 279; 3 Gy: 458 ± 237; 10 Gy: 107 ± 28 cells/hemi dentate gyrus; Fig 1G). On the other hand, when mice were subjected to electrical footshock stress (FSS) for 5 consecutive days at 8 weeks old, and proliferating cells were labeled with BrdU injection immediately afterwards, we did not observe any inhibition of neurogenesis at 8 to 12 weeks of age (Fig 1C, 1H). These results suggested that the stress paradigm employed in this study was sufficiently weak that it alone did not inhibit neurogenesis. Moreover, no synergistic effect of X-irradiation and FSS on inhibition of neurogenesis was observed when proliferating cells were BrdU-labelled immediately after FSS (Control: 188 ± 36; FSS: 190 ± 32; 10 Gy: 82 ± 19; 10 Gy + FSS: 74 ± 8 cells/hemi dentate gyrus; Fig 1D, 1H).

**Figure 2:**
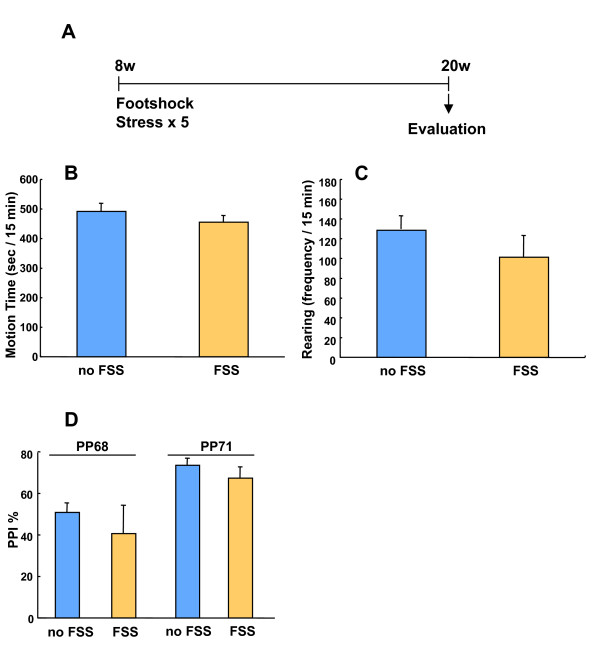
Behavior in the open field test (B, motion time; C, rearing), and PPI test (D) when measured at 20 weeks of age was not affected by the administration of FSS for 5 consecutive days at 8 weeks of age. n = 6.

Next, we examined whether the FSS technique employed in this study caused any behavioral effects. Mouse behavior was examined in the open field test and prepulse inhibition (PPI) test (Fig 2). No differences between FSS treated and untreated mice were observed (Fig 2B, motion time, no FSS: 491.1 ± 27.0; FSS: 454.8 ± 21.7 sec/15 min; Fig 2C, rearing, no FSS: 128.6 ± 7.7; FSS: 101.2 ± 16.1 frequency/15 min; Fig 2D, PPI, no FSS: 50.9 ± 4.5; FSS: 40.6 ± 13.6% in the case that prepulse was 68 dB, no FSS: 73.5 ± 3.4; FSS: 67.4 ± 5.3 in the case that prepulse was 71 dB). The results suggested that the stress procedure employed in this research was sufficiently weak that it alone did not cause any significant change in mouse behavior.

**Figure 3:**
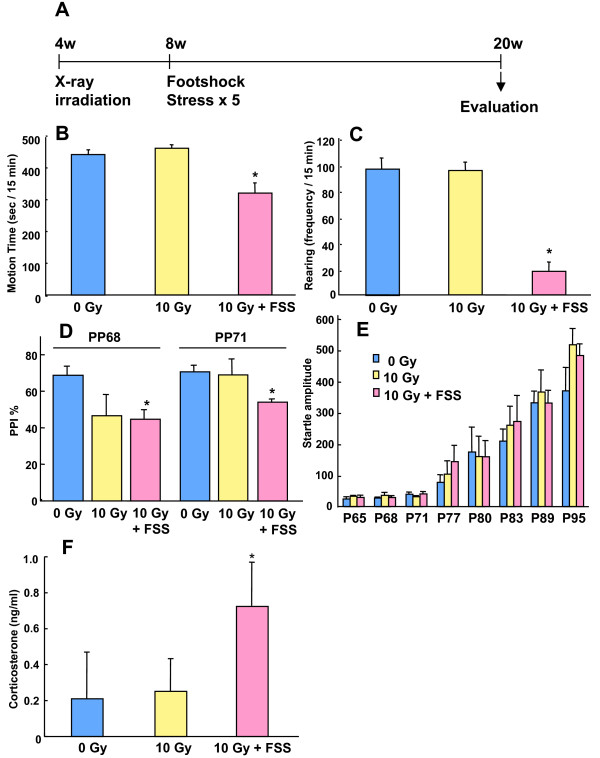
(A) Mice that were X-irradiated at 4 weeks of age, prior to experiencing footshock stress (FSS) were examined behaviorally at 20 weeks of age. (B, C) Activity in a novel environment was comparable between 0 and 10 Gy irradiated mice. However, mice irradiated with 10 Gy and subjected to FSS showed decreased motion time and rearing. (D) In the prepulse inhibition (PPI) test, 10 Gy plus FSS mice showed significant decreases in PPI following both 68 dB and 71 dB stimuli. (E) Startle response was not altered by X-irradiation or the combination of X-irradiation and FSS. Mice were placed in the conditioning chamber for 1 min and then presented with tones (1000 Hz and 3000 Hz, 3 sec in length) of increasing sound intensity. The interval between tones was 10 sec. The sound level required to elicit an orienting reflex was determined. (F) Corticosterone was increased significantly in 10 Gy plus FSS group. All data were expressed as mean ± S.E.M, n = 6–10.

Finally, we examined the effect of X-irradiation on behavior. Mice were X-irradiated at 4 weeks of age and were subjected to FSS at 8 weeks. Behavioral changes and serum corticosterone levels were then examined at 20 weeks. Mice receiving both X-irradiation and FSS showed decreased motion time (Fig 3B, 0 Gy: 442 ± 14; 10 Gy: 462 ± 10; 10 Gy + FSS: 321 ± 31 sec/15 min) and rearing (Fig 3C, 0 Gy: 97 ± 8; 10 Gy: 95 ± 6; 10 Gy + FSS: 18 ± 7 times/15 min) in the open field test. Moreover, X-irradiated mice showed PPI deficits (0 Gy: 70.3 ± 4.9; 10 Gy: 48.9 ± 8.7; 10 Gy + FSS: 47.5 ± 4.8% in the case that prepulse was 68 dB, 0 Gy: 72.5 ± 3.5; 10 Gy: 70.4 ± 8.7; 10 Gy + FSS: 58.3 ± 2.4% in the case that prepulse was 71 dB, Fig 3D) without any significant change in the startle response (Fig 3E). Furthermore, serum corticosterone levels were increased in the mice that experienced both X-irradiation and FSS (0 Gy: 0.21 ± 0.26; 10 Gy: 0.25 ± 0.18; 10 Gy + FSS: 0.72 ± 0.24 ng/ml, Fig 3F). X-irradiation alone had no effects on these behaviors. These findings suggest that the mice subjected to X-irradiation were more vulnerable to stress, and that this may resulte in behavioral changes such as decreased locomotor activity and PPI deficits.

## Discussion

In this study, we found that mice that experienced both X-irradiation and stress during adolescence had increased serum corticosterone levels and showed decreased locomotor activity and deficits in sensory gating in adulthood. These results suggest that hippocampal neurogenesis could be an important factor involved in biological vulnerability to the onset of psychiatric disorders.

In the present study, mice were X-irradiated at 4 weeks of age, which is thought to be the rodent equivalent of juvenile adolescence [13]. Previous reports have indicated that impairments in prenatal neurogenesis can have long-lasting effects, causing abnormal behavior later in life, during adulthood [12]. Our results suggest that postnatal neurogenesis, in addition to prenatal neurogenesis, may lower the vulnerability to disease and prevent its onset.

Here, X-irradiation was used to inhibit postnatal neurogenesis in the mouse hippocampus. Decreased neurogenesis, achieved via irradiation, causes impaired long-term potentiation (LTP) [14], decreased efficacy of anti-depressive drugs [15], and disabilities in recognition [16]. In addition to inhibition of neurogenesis, X-irradiation causes other problems such as an inflammatory response [17] and blood vessel destruction [16]. At present, we cannot rule out the possibility that these factors might also be involved in the observed responses to stress. Further studies, perhaps involving gene knockout mice with abnormal hippocampal neurogenesis, are required in order to resolve this issue.

Genetic factors for psychiatric disorders have been identified using linkage analysis (Ross et al., 2006). For example, DISC1 is a gene that is disrupted in schizophrenia patients and is known to be causative factor for schizophrenia and bipolar disorder [18]. It was reported recently that downregulation of DISC1 in hippocampal stem/progenitor cells caused abnormal cell positioning, alterations in dendritic arborization in newly-formed neuron, and enhanced neuronal activity in adult mice [19]. The role of DISC1 in neurogenesis suggests that not only decreased neurogenesis but also abnormal neurogenesis can alter the formation of neural circuits in the hippocampus. Although only weak stress, in the form of electrical footshock, was employed in the present study, stronger stress such as restraint stress has been previously shown to inhibit hippocampal neurogenesis [20]. Such findings suggest that postnatal neurogenesis in the hippocampus could be involved, as either a genetic factor or an environmental factor, in the biological vulnerability-stress hypothesis.

In the present study, abnormal behaviors such as decreased locomotor activity in the open field and sensory-gating deficits in the PPI test were observed in adult mice subjected to both X-irradiation and stress during adolescence (Fig. 3). Mouse behavior was also examined using the forced swim test, elevated plus maze test and tone-fear conditioning test. However, no significant effects were observed in these additional tests (data not shown), suggesting that any symptoms that did occur did not amount to depression or anxiety. PPI is a well-known physiological phenotype that is observed in human schizophrenic and bipolar patients. It is thought that sensory gating deficits in the PPI test in rodents are analogous to the cognitive deficits and other symptoms seen in human schizophrenia patients. However, in contrast to our results, the inhibition of neurogenesis through X-irradiation has been reported to enhance working memory, which is often compromised in schizophrenic patients [21]. As such, abnormalities in recognition ability should be examined carefully in future studies of abnormal neurogenesis.

A number of studies have shown that stressors experienced during adolescence can have long-lasting and profound consequences for the future behavioral and psychological function of an individual [6]. Impaired prenatal neurogenesis causes abnormal behavior later in life, during adulthood [12]. These studies suggest that the effects of stress and decreased neurogenesis during adolescence can be long-lasting. In fact, our data showed that X-irradiated mice subjected to stress during adolescence exhibited abnormal behavioral phenotypes in adulthood. Especially in the field of schizophrenia research, it is hypothesized that some abnormalities in the postnatal development of forebrain neural circuits are thought to affect the onset of schizophrenic symptoms, even in late-onset cases where the incidence of schizophrenia occurs long after the stressful stimulus, which might occur during adolescence [2]. We consider the experimental model employed in this study to be a useful disease model, reflective of at least a subset of schizophrenia cases. The results generated from this model suggest that postnatal neurogenesis could be a critical biological process involved in the onset of schizophrenic symptoms.

## Conclusion

Our research suggests that decreased neurogenesis during adolescence is accompanied by an increase in the individual's vulnerability to the development of behavior related to psychiatric disorders. A further understanding of the mechanisms underlying postnatal neurogenesis in the hippocampus during adolescence could help to elucidate the mechanisms governing biological vulnerability to psychiatric disease.

## Methods

### Animals

All procedures involving the use of mice complied with the guidelines of the National Institute of Health and were approved by the Animal Care and Use Committee of Mitsubishi Kagaku Institute of Life Sciences. Male C57 Black 6J mice (4 weeks old) were obtained from CLEA Japan Inc. (Tokyo, Japan).

### X-ray irradiation

Mice were anesthetized using an intraperitoneal injection of pentobarbital solution (50 mg/kg of body weight) and fully-anesthetized mice were irradiated in a Hitachi X-irradiation apparatus (MBR-1505R, HITACHI, Tokyo, Japan) with shielding their body with lead.

### Footshock stress

The conditioning chamber consisted of an observation box (32 × 22 × 22 cm) made of clear and gray vinylchloride plates as used previously [22,23]. The chamber's floor consisted of 28 stainless steel rods (0.4 cm diameter, spaced 1 cm apart) through which footshocks were delivered by a scrambled-footshock generator (Muromachi, SGS 002). The chamber was placed in a light- and sound-attenuating room where external noise was greatly reduced (-35 dB at 500 Hz). The chamber was cleaned with ethanol (99.5%) and dried with a hair drier before and after the occupancy of each mouse. A video camera placed in front of the chamber recorded the behavior of each mouse. A controller for conditioning was operated by a remote switch placed in an adjacent room. Each mouse was placed in the chamber for 5 min and received five footshocks (1.0 mA intensity, 2.0 sec duration, 30.0 sec interval).

### Cell proliferation, cell death and neurogenesis

Cell proliferation and cell fate determination were examined as described previously [23,24]. To examine cell proliferation, mice were injected intraperitoneally with BrdU (Sigma) in 0.9% saline solution (50 mg/kg of body weight) and were sacrificed with an overdose of pentobarbital (200 mg/kg body weight) 2 hours later (Fig 1A). To examine the number of newly formed neurons, BrdU was administered daily (50 mg/kg body weight, three times per day for three days) and sacrificed 4 weeks later (Fig 1B). Animals were perfused transcardially with 0.9% saline followed by 4% paraformaldehyde (PFA) in phosphate buffered saline (PBS). Brains were dissected out and postfixed (4% PFA in PBS) for 2 hours at 4°C, incubated in 30% sucrose in PBS overnight at 4°C, and then immersed in dry ice powder. A cryostat was used to collect sagittal sections of 14 μm thickness. Sections were boiled for 10 min, treated with 2 M HCl at 37°C for 30 min and washed three times with PBS and Tris-buffered saline containing 0.1% Tween20 (TBST). Sections were blocked with 5% non-fat milk in TBST at room temperature for 30 min, then were incubated with blocking solution containing rat monoclonal anti-BrdU (1:120; Abcam). Newly-formed neurons were examined using a mouse monoclonal anti-NeuN (1:300; Chemicon) antibody. Sections were then reacted with anti-rat IgG-FITC and anti-mouse IgG-Rhodamine. Labeled cells were quantified using three consecutive serial sections at 13 section intervals. BrdU-positive cells were counted using a x40 objective lens (BX41 OLYMPUS) in a group blind manner (Supplemental Fig 1). Numbers of BrdU-positive and NeuN-positive cells were obtained by multiplying the counts by 13/3. To estimate cell death, an Apoptag fluorescence *in situ *apotosis detection kit (Chemicon) was employed, and apoptotic cells were counted in a manner similar to that employed in the BrdU experiments.

### Open field test

General activity was measured in an open field box (50 × 50 × 30 cm) constructed from gray vinylchloride plates [22,23]. The apparatus was placed in another sound-attenuating room where external noise was greatly reduced (-45 dB at 500 Hz). Two pairs of 7 × 7 array infrared photosensors were attached to the outer wall, equally spaced in lower and upper rows, 1.5 and 16 cm above the floor, respectively. The lower row of photocells was used to measure locomotor activity and the upper row to detect rearing behavior. The sensor state was sampled every 0.5 sec. A computer recorded the number of horizontal photobeam interruptions caused by animal movement.

### Auditory startle and Prepulse Inhibition of acoustic startle

Mice were tested in a startle chamber (SR-lab System, San Diego Instruments, San Diego, CA) positioned within a soundproof cabinet in a sound-attenuating room according to standard methodology [23]. A constant background noise of 65 dB was presented throughout the test. To measure prepulse inhibition (PPI) scores, mice were presented with a 68 or 71 dB prepulse (pp68 or pp71; 20 ms in length) followed by a 105 dB pulse (40 ms in length) 100 ms later. The percent prepulse inhibition of the startle response was calculated using the following formula: 100-((SRPP/SR) × 100) where SR denotes the startle response to the pulse stimulus, and SRPP denotes the startle response to the pulse when preceded by the prepulse stimulus.

### Auditory threshold test

Mice were placed in the conditioning chamber for 1 min and then presented with tones (1000 Hz and 3000 Hz; 3 sec in length) of increasing sound intensity [23]. The interval between tones was 10 sec. The sound intensity required to elicit an orienting reflex to the sound source was determined.

### Corticosterone measurement

Anesthesia with isoflurane was induced in a sealed chamber (Neuroscience, Tokyo, Japan) and maintained via a mask while serum samples were taken. Serum sample was taken between 10:00 am and 2:00 pm. Each serum sample was centrifuged for 20 min at 14,000 rpm at room temperature and the clear supernatant was stored at -80°C until assay. The corticosterone content of the thawed serum sample was measured using an enzyme immunoassay kit (Cayman, Michigan).

### Statistical analysis

The experimental data were analyzed using a one-way or two-way analysis of variance (ANOVA). Comparisons between pairs of groups were carried out using Student's *t*-test. Values of P < 0.05 were considered to be statistically significant. All values in the text and figure legends are expressed as mean ± S.E.M.

## Abbreviations

FSS: footshock stress; HPA-axis: hypothalamus-pituitary-adrenal cortex-axis; PVN: paraventricular nuclei; CRH, corticotrophin releasing hormone; BrdU: 5-bromodeoxy uridine; PFA: paraformaldehyde; PBS: phosphate buffered saline; TBST: Tris-buffered saline containing 0.1% Tween20; PPI: prepulse inhibition.

## Competing interests

The authors declare that they have no competing interests.

## Authors' contributions

FH and KI conceived and designed the experiment. NT, AM and FH performed the experiments. FH and KI wrote the paper.

## Supplementary Material

Additional file 1 A representative micrograph of cells counted in the experiments shown in Figure 1. (A) 0 Gy and (B) 10 Gy samples from cell proliferation experiment (Fig 1E). (C) 0 Gy and (D) 10 Gy samples from the cell differentiation experiment (Fig 1G). (E) 0 Gy and (F) 10 Gy samples from the cell death experiment (Fig 1F). The counted cells are indicated using arrowheads. Click here for file
