# Supplementary figures and images for: Decreased postnatal neurogenesis in the hippocampus combined with stress experience during adolescence is accompanied by an enhanced incidence of behavioral pathologies in adult mice
Source: Mol Brain. 2008 Dec 17;1:22. doi: 10.1186/1756-6606-1-22 (PMC2628657; doi:10.1186/1756-6606-1-22)

## Slide 1
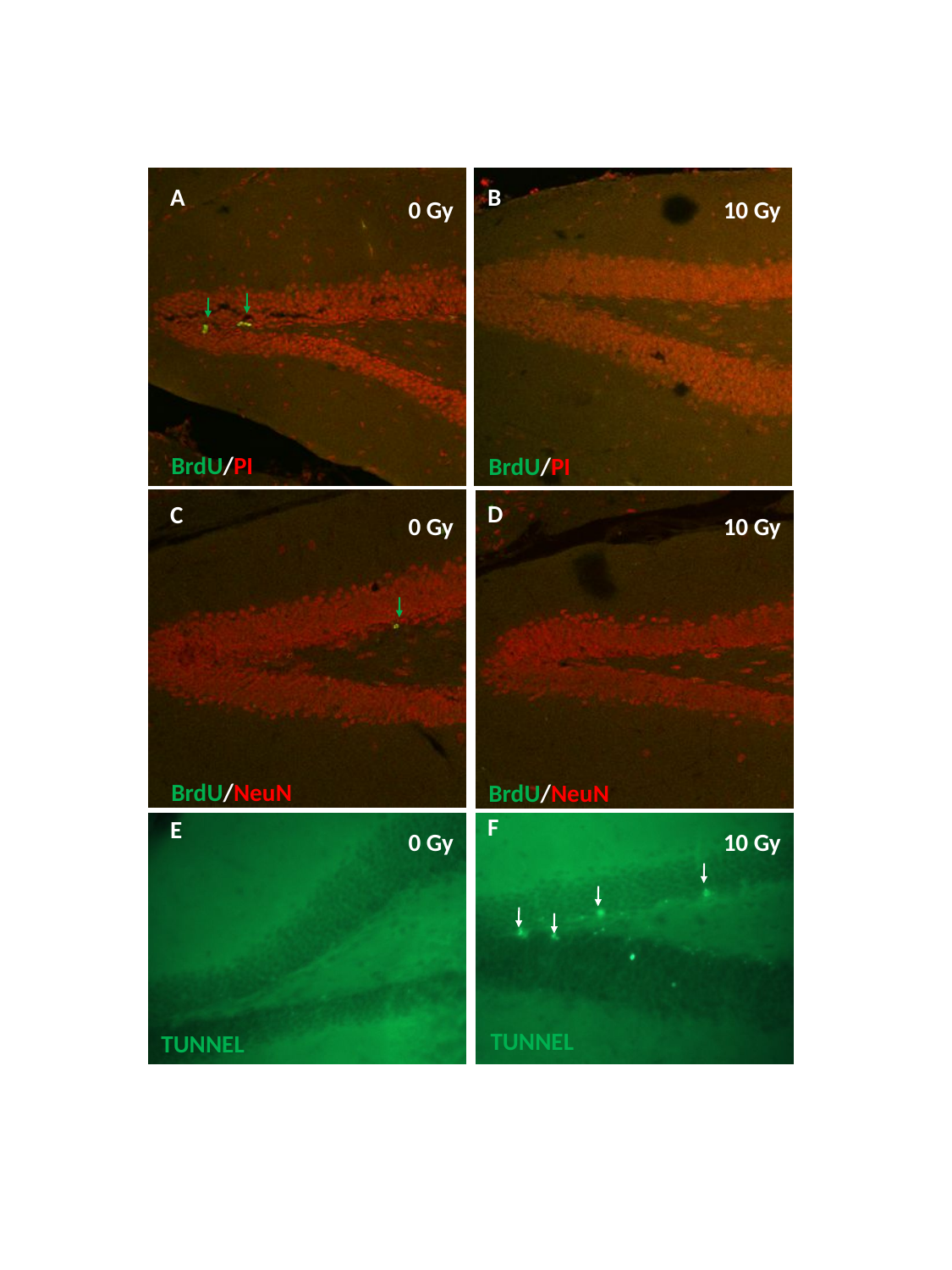

A
B
0 Gy
10 Gy
BrdU/PI
BrdU/PI
D
C
0 Gy
10 Gy
BrdU/NeuN
BrdU/NeuN
F
E
0 Gy
10 Gy
TUNNEL
TUNNEL

Supplement: Additional file 1 — A representative micrograph of cells counted in the experiments shown in Figure 1. (A) 0 Gy and (B) 10 Gy samples from cell proliferation experiment (Fig 1E). (C) 0 Gy and (D) 10 Gy samples from the cell differentiation experiment (Fig 1G). (E) 0 Gy and (F) 10 Gy samples from the cell death experiment (Fig 1F). The counted cells are indicated using arrowheads. [file 1756-6606-1-22-S1.ppt]
